# Baseline characteristics of survivors, primary care clinicians, and oncologists in a randomized trial of a shared care, self-management intervention for cancer survivors

**DOI:** 10.1007/s00520-025-09774-2

**Published:** 2025-08-02

**Authors:** Maria Berkeley, Harold Urman, Janet Lee, Yi Xiao, Betty Ferrell, Anne Reb, Marc Debay, Stuart Miller, Linda Lucero, Anne Charity Hudley, Virginia Sun

**Affiliations:** 1 Department of Population Sciences, City of Hope, 1500 East Duarte Road, Duarte, CA 91010 USA; 2 Vital Research, Los Angeles, CA USA; 3 Department of Family Medicine, University of California at Riverside, Riverside, CA USA; 4 Patient Partner, Studio City, CA USA; 5 Patient Partner, Las Vegas, NV USA; 6 Patient Partner, Graduate School of Education, Stanford University, Stanford, CA USA; 7 Department of Surgery, City of Hope, Duarte, CA USA

**Keywords:** Cancer survivor, Telehealth, Self-management, Care coordination, Communication

## Abstract

**Purpose:**

Lung and colorectal cancer (CRC) survivors experience late and long-term treatment effects and challenges with navigating care. Few evidence-based interventions exist to support survivor needs. This paper describes participant recruitment and pre-randomization baseline characteristics and outcomes from a survivorship self-management intervention trial in lung and CRC.

**Methods:**

Baseline outcome measures were collected from survivors, primary care providers (PCP), and oncologists. Enrolled participants were survivors of lung or CRC, were 4–6 months post-treatment completion, age 18 or older, and could read and understand English. Survivor outcome measures included geriatric assessment, quality of life (QOL), communication, knowledge, and self-efficacy. PCP and oncologist outcome measures assessed perceived knowledge, communication, and care coordination regarding survivorship care.

**Results:**

The trial completed accrual over 4 years and enrolled 404 participants across 15 clinical practice sites in Southern California. At baseline, most survivors reported high (mean = 71.11/100) levels of physical functioning and social support but moderate (mean = 53.29/100) levels of social interactions. QOL scores were low for emotional and functional well-being, with survivors of lung cancer reporting lower physical well-being (20.92/28) and total QOL (101.1/136). PCPs and oncologists reported minimal problems with exchanging information or transferring care in a timely manner. Survivors reported challenges with timely care, appointments, and support managing treatment effects.

**Conclusions:**

Baseline characteristics illustrate persistent challenges in survivor QOL and perceived quality of care coordination and communication among survivors, oncologists, and PCPs.

**Implications for Cancer Survivors:**

Opportunities for improvements in cancer survivorship care delivery exist and will ultimately support survivors’ QOL and outcomes.

## Introduction

Many Americans are living longer than ever before and with more comorbidities due to advancements in medicine and science [1]. Cancer survivors make up a large subset of this group, with approximately 78% being over the age of 60 in the USA [2]. In particular, lung and colorectal cancer (CRC) are among the top 10 most common cancers and have the highest survivorship prevalence [3]. These survivors experience unique and complex needs, and late and long-term effects are common, meaning that our reactive healthcare system is not designed to optimally serve them. Lung and CRC survivor post-treatment needs vary from low to high and many are non-cancer specific. In addition, survivors variably receive follow-up care from either their primary care provider (PCP), or oncologist, or survivorship care is shared between the two. Lack of clarity about the specific role of each clinician contributes to disruptions in care coordination and communication, fragmenting care [4].

Currently, there are few evidence-based survivorship care interventions for lung and CRC. A National Academy of Medicine workshop found that current models of survivorship care continue to fail in meeting the needs of survivors, despite decades of progress in survivorship care research [5]. Additionally, there is a need to develop and test comprehensive strategies of survivorship care that can support primary care and oncology to provide quality survivorship care [6]. A promising strategy is to leverage shared care models where oncologists and PCPs receive support/information on their roles and responsibilities in caring for cancer survivors while supporting self-management skill-building for survivors. Self-efficacy, or self-confidence, is essential for patients to successfully navigate and manage post-treatment survivorship care. There is evidence that self-management coaching improves patient confidence, and physical and psychosocial health, and healthcare use, particularly in the context of chronic conditions such as cancer [7]. The purpose of this paper is to describe recruitment and pre-randomization baseline sociodemographic characteristics along with outcomes from a self-management intervention trial for survivors with lung and CRC.

## Materials and methods

This study was a two-arm randomized controlled trial, conducted at a National Cancer Institute (NCI)-designated comprehensive cancer center and affiliated practice sites in the greater Los Angeles County and Orange County area of Southern California. The US NCI-designated comprehensive cancer centers are recognized for their leadership in transdisciplinary and innovative cancer research, as well as their commitment to rigorous methods in both cancer prevention and treatment [8]*.* Survivors who participated in the study were determined to have met the following inclusion criteria: (1) survivor of lung or CRC 4–6 months post-treatment completion; (2) history of stage I–III disease; (3) age 18 years or older; (4) able to read and understand English. For survivors of lung cancer, those who were receiving immunotherapies or targeted therapies for primary treatment, adjuvant treatment, or maintenance treatments were also eligible for the study. This criterion was added to the study to accommodate the needs of survivors living long-term with metastatic/advanced cancers as well as to reflect the changing landscape of treatment for lung cancer. Written informed consent was obtained and baseline measures were collected using paper surveys or electronically using REDCap. Alternative methods of consent included in person, telephone, mail, and electronic mail. The study was approved by the cancer center’s Institutional Review Board (IRB).

During the course of the study, researchers implemented several strategies to enhance participant recruitment. Four patient partners were included in the study and supported the designing, refining, and troubleshooting of all recruitment strategies and challenges. This approach allowed for feedback from the survivor’s perspective and ensured that the study’s research strategy was patient-centered. Study staff worked closely with oncologists across the clinical practice sites and identified engagement and referral strategies based on oncology care team workflows and preferences. The core research team met bi-weekly to monitor accrual progress, and a monthly accrual graph was sent via email to all participating clinicians to measure engagement. Finally, one-page flyers were created and placed on the walls of clinician workrooms with reminders of the study purposes, inclusion criteria, and study staff contact information for referrals. All methods of study advertisement, including brochures and flyers, were approved by the IRB for recruitment purposes.

Potential participants were screened and recruited by study staff who worked closely with oncology care teams throughout the cancer center’s clinical practice sites in Southern California. Prior to enrollment, all survivor participants provided signed informed consent. Following informed consent, all survivor participants completed baseline outcome measures. Survivor participants were also asked if they have a PCP (physician, advanced practice provider, etc.). Baseline questionnaires were sent to the identified PCPs and the survivor’s treating oncologist. Signed informed consents were waived for the PCPs and oncologists, and they were made aware that completion of outcome measures served as consent to participate in the study. Following the outcome measures completion, survivors were randomized into either the intervention group or the attention control group, using a stratified and blocked randomization method based on disease type and stage. The survivor participant’s PCPs and oncologists were randomized to the same group as the survivor participant.

The intervention design and rationale has been previously described [9]. Briefly, participants randomized to the survivorship self-management intervention group received eight telehealth (videoconferencing) sessions delivered by advanced practice nurses (APRNs) over a period of 8 months. APRNs were selected because they possess the training and skills to address survivorship care issues. Furthermore, they routinely practice alongside oncologists and PCPs in care settings [10]. The first five sessions were delivered over 4 months and addressed physical, psychological, social, and spiritual well-being and lifestyle behavior change. The last three sessions were delivered over a 3-month period and were designed as maintenance sessions to support self-management and review additional topics. Self-management coaching strategies included goal setting, identifying challenges in meeting goals, and action planning to overcome challenges [10].

The APRNs facilitated care coordination and communication between patients, oncologists, and PCPs. Specifically, the APRNs provided a personalized, one-page summary survivorship care plan to the PCPs. The APRNs noted all intervention session encounters in the Electronic Health Record (EHR), making the information available to all oncology care teams. Personalized, disease-specific care plans were provided to each participant, and an intervention resource manual with session content and other resources were also provided to participants. Participants randomized to the attention control group received the NCI handbook, “Facing Forward: Life After Cancer Treatment.” The booklet contains practical tips and guidelines for managing health and relationships and was created by survivors and clinicians specifically for survivors. They also received eight telehealth sessions with Attention Control nurses. During the sessions, the nurses encouraged participants to review the NCI handbook throughout the study and made themselves available to answer any questions on the handbook if needed. There were no study-related interactions with the PCPs and oncologists of attention control group participants.

### Outcome measures

All baseline data from survivors, PCPs, and oncologists were completed following informed consent and prior to randomization. Survivors completed a Comprehensive Geriatric Assessment to evaluate medical, social, and functional limitations [11, 12]. All participants, regardless of age, completed the Geriatric Assessment. Survivor experiences with cancer care, specifically care coordination, communication, and knowledge, were assessed by The Care Coordination – Consumer Assessment of Healthcare Providers and Systems (CAHPS) Cancer Care Survey [13]. Survivors’ knowledge and self-efficacy related to prior disease, treatments, and future survivorship care needs were assessed by the Confidence in Survivorship Information (CSI) tool [14]. Survivor’s perceived quality of communication was evaluated by the Patient-Centered Communication (PCC) Survey [15]. Finally, survivor Quality of Life (QOL) was assessed by the Functional Assessment of Cancer Therapy-Lung (FACT-L) or the Functional Assessment of Cancer Therapy-Colorectal (FACT-C) [16 17]. The tools are cancer-specific and contain questions on the physical, social/family, emotional, and functional well-being domains as well as disease-specific symptom indexes [13–18]. PCP and oncologist outcome measures included selected survey items from the Survey of Physician Attitudes Regarding the Care of Cancer Survivors (SPARCCS) Study [19]. Two distinct surveys were developed: one for oncology specialists and one for PCPs [19]. The study used selected items from the SPARCCS surveys that assessed perceived roles, communication, and care coordination with regard to survivorship care. Questions on knowledge of survivorship care were only completed by PCPs, and practice and select sociodemographics were collected from PCPs only.

### Statistical analysis

Sample size calculation was performed using G*Power [20]. To achieve the desired power of 0.80 to detect a medium (0.25) or large (0.40) effect size with a conservative alpha of 0.01, a sample size of 330 patients was needed. To account for a 20% attrition rate, a total of 404 participants were enrolled. To analyze the baseline data for survivors, PCPs, and oncologists, descriptive statistics such as means, standard deviations, frequencies, and percentages were calculated for all outcome measures using SPSS software (v.24 or higher). For survivors, means and standard deviations were calculated for continuous demographic variables (e.g., age) or outcomes with scaled scores (e.g., Comprehensive Geriatric Assessment, survivors’ experience with cancer care, confidence about survivorship information, patient-centered communications, and quality of life) for overall participants as well as for each diagnosis group (lung and CRC). Frequencies and percentages were calculated for categorical socio-demographic variables (e.g., gender, race, education), as well as for overall participants and by diagnosis group. The frequencies of missing data were included in the analysis.

For PCPs, all outcome measures of practice characteristics and survey items measuring confidence about knowledge of cancer survivorship care were categorical. Therefore, frequencies and percentages were calculated, including the frequency of missing responses. The select survey items from the SPARCCS completed by both PCPs and oncologists measuring perceived roles, communication, and care coordination with regard to survivorship care were also categorical in nature. To understand how PCPs and oncologists were similar or different in their perceptions of these outcomes, a comparative analysis using the percentage distribution of responses to survey items was conducted. For example, the percentage of PCPs who indicated they always/almost *received* a cancer follow-up care summary was compared to the percentage of oncologists who indicated they always/almost always *provided* a cancer follow-up care summary.

## Results

The trial enrolled 404 participants between April 2020 and June 2024. Most participants (*n* = 298) were enrolled at the main cancer center campus with smaller numbers enrolled at other 15 network practice sites located in Los Angeles, Orange, Riverside, and San Bernardino Counties. A total of 15 clinical practice sites located in the three Counties contributed to study enrollment. This included the clinical practice site in Antelope Valley, Southern California’s high desert area. The trial continued with accrual through the beginning of the COVID-19 pandemic in 2020 and subsequent surges.

Of the 404 participants enrolled, 11 participants attritioned before completing baseline measures, with 393 participants that completed baseline measures (Table 1). The mean age for all survivors was 63 years, with a mean age of 66 for survivors with lung cancer and 59 for survivors with CRC. Over half of the participants were women (56%). The majority of participants were White (66%), followed by 15% Asian, 15% Hispanic/Latinx, and 4% Black/African American. More than half of all participants (52%) reported few disease-related symptoms and were able to carry on with normal activity. Over half of the individuals enrolled (51%) had stage III or IV, disease and nearly 4% were current smokers. Most survivors (83%) had a designated PCP at the time of consent, and 20% were followed by an oncologist in combination with another specialist.

**Table 1:** Baseline survivor and primary care provider characteristics

Survivor characteristics	Overall (*N* = 393)	Lung cancer (*N* = 213)	Colorectal cancer (*N* = 180)
Age, mean (SD)	62.9 (12.4)	66.3 (10.6)	58.9 (13.1)
Gender			
Female	221 (57.6%)	131 (63.0%)	90 (51.1%)
Male	163 (42.4%)	77 (37.0%)	86 (48.9%)
*Missing/refused to answer*	9	5	4
Race			
White	258 (65.6%)	139 (65.3%)	119 (66.1%)
Asian	57 (14.5%)	38 (17.8%)	19 (10.6%)
African American	15 (3.8%)	8 (3.8%)	7 (3.9%)
American Indian or Alaska Native	4 (1.0%)	0 (0.0%)	4 (2.2%)
More than one race	10 (2.5%)	6 (2.8%)	4 (2.2%)
Native Hawaiian or Pacific Islander	2 (0.5%)	2 (0.9%)	0 (0.0%)
Other	20 (5.1%)	6 (2.8%)	14 (7.8%)
*Missing/refused to answer*	27 (6.9%)	14 (6.6%)	13 (7.2%)
Ethnicity			
Hispanic	58 (14.8%)	20 (9.4%)	38 (21.1%)
Non-Hispanic	313 (79.6%)	180 (84.5%)	133 (73.9%)
*Missing/refused to answer*	*22 (5.60%)*	*13 (6.1%)*	*9 (5%)*
Education			
(Some) Graduate school	111 (29.0%)	60 (28.7%)	51 (29.3%)
(Some) College	207 (54.0%)	111 (53.1%)	96 (55.2%)
Vocational school/completed high school/GED	55 (14.4%)	35 (16.7%)	20 (11.5%)
Did not complete high school	10 (2.6%)	3 (1.4%)	7 (4.0%)
*Missing/refused to answer*	*10*	*4*	*6*
Marital status			
Married/partnered	265 (70.9%)	145 (71.4%)	120 (70.2%)
Divorced/separated	41 (11.0%)	27 (13.3%)	14 (8.2%)
Single	37 (9.9%)	13 (6.4%)	24 (14.0%)
Widowed	31 (8.3%)	18 (8.9%)	13 (7.6%)
*Missing/refused to answer*	*19*	*10*	*9*
Employment status			
Full-time	109 (28.3%)	40 (19.1%)	69 (39.2%)
Part-time	40 (10.4%)	22 (10.5%)	18 (10.2%)
Retired	168 (43.6%)	116 (55.5%)	52 (29.5%)
Disabled	20 (5.2%)	11 (5.3%)	9 (5.1%)
Unemployed	45 (11.7%)	19 (9.1%)	26 (14.8%)
Other	3 (0.8%)	1 (0.5%)	2 (1.1%)
*Missing/refused to answer*	*8*	*4*	*4*
Household income			
Less than $50,000	91 (25.1%)	49 (25.3%)	42 (25.0%)
$50,001 to $100,000	105 (29.0%)	57 (29.4%)	48 (28.6%)
Greater than $100,000	166 (45.9%)	88 (45.4%)	78 (46.4%)
*Missing/refused to answer*	*31*	*19*	*12*
Smoking status			
A current smoker	15 (3.94%)	9 (4.35%)	6 (3.45%)
Quit less than 6 months ago	11 (2.89%)	10 (4.83%)	1 (0.57%)
Quit more than 6 months ago	148 (38.85%)	103 (49.76%)	45 (25.86%)
Never a smoker	207 (54.33%)	85 (41.06%)	122 (70.11%)
*Missing/refused to answer*	*12*	*6*	*6*
Living with smoker(s)			
No	361 (94.01%)	191 (91.83%)	170 (96.59%)
Yes	23 (5.99%)	17 (8.17%)	6 (3.41%)
*Missing/refused to answer*	*9*	*5*	*4*
Disease stage			
I	88 (22.4%)	46 (21.6%)	42 (23.3%)
II	94 (23.9%)	28 (13.1%)	66 (36.7%)
III	120 (30.5%)	51 (23.9%)	69 (38.3%)
IV	91 (23.2%)	88 (41.3%)	3 (1.7%)
Have a primary care clinician at consent			
No	66 (16.79%)	36 (16.9%)	30 (16.67%)
Yes	327 (83.21%)	177 (83.1%)	150 (83.33%)
Have other medical specialists			
No	317 (80.66%)	155 (72.77%)	162 (90%)
Yes	76 (19.34%)	58 (27.23%)	18 (10%)
Primary care provider characteristics (*N* = 75)			
Type of provider, *N* (%)			
Physician			63 (85.14%)
Nurse practitioner			3 (4.05%)
Physician assistant			4 (5.41%)
Other			4 (5.41%)
*Missing/refused to answer*			*1*
Years of practice, *N* (%)			
0–9 years			9 (12.33%)
10–14 years			6 (8.22%)
15 + years			58 (79.45%)
*Missing/refused to answer*			*2*
Practice background, *N* (%)			
Family practice			41 (56.16%)
Internal medicine			30 (41.1%)
General practice			2 (2.74%)
*Missing/refused to answer*			*2*
Practice setting, *N* (%)			
Full- or part-owner of a physician practice			40 (54.05%)
Employee of a physician-owned practice			11 (14.86%)
Employee of a large medical group/health care system/HMO			19 (25.67%)
Employee of a university hospital or clinic			2 (2.7%)
Employee of a hospital or clinic not associated with a university (e.g., community health clinics)			2 (2.7%)
*Missing/refused to answer*			*1*
Number of cancer patients seen in practice, *N* (%)			
25 or fewer			58 (81.69%)
26–50			10 (14.08%)
51–75			3 (4.23%)
*Missing/refused to answer*			*4*
Age			
30–49			22 (31.88%)
50–69			41 (59.42%)
70 +			6 (8.7%)
*Missing/refused to answer*			*6*
Race, *N* (%)			
White			40 (57.97%)
Asian			16 (23.19%)
African American			1 (1.45%)
Native Hawaiian or Pacific Islander			1 (1.45%)
American Indian or Alaska Native			1 (1.45%)
Other			10 (14.49%)
*Missing/refused to answer*			*6*
Ethnicity, *N* (%)			
Non-Hispanic			61 (88.41%)
Hispanic			8 (11.59%)
*Missing/refused to answer*			*6*

Most PCPs (Table 1) were physicians (85%) and advanced practice professionals (9%) and 82% reported providing current care for 25 or fewer survivors with a cancer diagnosis at their practice. The majority of PCPs (58%) self-reported as White, had 15 years or more of professional experience (79%), and were between the ages of 40 and 69 (80%).

Survivor outcomes are presented in Table 2. For baseline select geriatric assessment domains, overall physical functioning scores were moderately high (mean = 71.1/100), and most participants were able to complete instrumental activities of daily living independently (mean = 13.1/14). Most individuals could independently manage their day-to-day activities with minimal interference from their illness. Overall, survivors also reported high (mean = 4.29/5) levels of social support but moderate (mean = 53.29/100) levels of social interaction.

**Table 2:** Baseline outcome measure scores for survivors

	**Overall (*N* = 383)**	**Lung cancer (*N* = 207)**	**Colorectal cancer (*N* = 176)**
**Comprehensive geriatric assessment, mean (SD)**			
**SF-36 Physical Functioning **[1]	71.11 (26.48)	65.05 (27.68)	78.31 (23.07)
**MOS Social Support, Tangible Support Subscale **[2]	4.34 (0.92)	4.36 (0.84)	4.31 (1.01)
**MOS Social Support, Emotional/Information Support Subscale **[2]	4.29 (0.93)	4.30 (0.91)	4.28 (0.95)
**OARS IADL Summary Score **[3]	13.1 (1.65)	12.82 (1.85)	13.43 (1.29)
**Number of IADL Activities that can be performed independently **[4]	6.05 (1.61)	5.82 (1.72)	6.32 (1.44)
**OARS Physical Health—Illnesses interference with activities **[5]	1.67 (0.53)	1.69 (0.52)	1.65 (0.54)
**OARS Physical Health—Number of Illnesses **[6]	1.75 (1.63)	2.02 (1.72)	1.44 (1.46)
**OARS Social Activities **[7]	53.29 (21.87)	51.76 (21.8)	55.12 (21.87)
**Survivor experiences with cancer care, mean (SD) **[8]			
**Getting timely appointments, care, and information**	56.88 (38.63)	56.73 (38.18)	57.06 (39.27)
**Cancer care team use of information to coordinate patient care**	63.85 (35.88)	65.63 (35.75)	61.77 (36.03)
**Cancer care team supports survivor in managing the effects of cancer and treatment**	53.13 (29.46)	52 (30.73)	54.46 (27.91)
**Cancer care team is available to provide information when needed**	69.18 (35.27)	67.79 (36.69)	70.83 (33.55)
**Involvement of family members and friends in discussion of care, *N* (%)**			
**Yes, definitely**	71 (18.5%)	34 (16.4%)	37 (21%)
**Yes, somewhat**	65 (17%)	32 (15.5%)	33 (18.8%)
**No**	247 (64.5%)	141 (68.1%)	106 (60.2%)
**Confidence in survivorship information **[9]			
**Confidence in knowledge of past cancer diagnostic and treatment details**	1.73 (0.39)	1.73 (0.4)	1.73 (0.38)
**Confidence in knowledge about treatment, prevention of future disease, access to resources, familial risk of cancer**	1.01 (0.6)	1 (0.62)	1.03 (0.58)
**Patient-centered communication **[10]	68.43 (34.71)	69.32 (34.4)	67.39 (35.14)
**Quality of life **[11, 12]			
**Physical well-being**	NA	20.92 (6.23)	22.82 (4.48)
***Missing/refused to answer***	NA	*0*	*1*
**Social/family well-being**	NA	22.92 (4.95)	22.55 (5.4)
**Emotional well-being**	NA	17.86 (4.3)	18.97 (4.28)
***Missing/refused to answer***	NA	*1*	*0*
**Functional well-being**	NA	18.75 (6.3)	20.4 (5.89)
**Disease-specific subscale**	NA	20.73 (4.25)	20.94 (4.66)
**Total quality of life**	NA	101.1 (19.15)	105.55 (18.27)
**Total Trial Outcome Index**	NA	60.41 (14.1)	64.03 (12.25)

[1] *SF-36*, 36-Item Short Form Survey. Physical functioning subscale score range 0–100. Higher scores indicate better physical functioning. [2] *MOS*, Medical Outcomes Study Social Support Survey. Score range 1–5. Higher scores indicate higher level of support. [3] *OARS IADL*, Older Americans Resources and Services Instrumental Activities of Daily Living. Score range 0–14. Higher scores indicate more independence. [4] Score range 0–8. Higher scores indicate high function/independent. [5] *OARS*, Older Americans Resources and Services Physical Health Section. Score range 1–6. Higher scores indicate higher impairments. [6] *OARS*, Older Americans Resources and Services number of illnesses (0–14). [7] *OARS*, Older Americans Resources and Services Social Activities. Score range 0–100. Higher scores indicate greater level of social interactions. [8] Consumer Assessment of Healthcare Providers and Systems (CAHPS) Cancer Care Survey. Score range 0–100. Higher scores indicate better rating of care. [9] *CSI*, Confidence in Survivorship Information Tool. Score range 0–2. Higher scores indicate more confidence. [10] Score range 0–100. Higher scores indicate better perceived communication. [11] *FACT-L*, Functional Assessment of Cancer Therapy-Lung. Physical well-being, Social/Family well-being, Functional well-being and Lung Cancer disease-specific subscale score range 0–28. Emotional well-being subscale score range 0–24. Total FACT-L Quality of Life score range 0–136. Total FACT-L trial outcome index score range 0–84. Higher scores indicate better quality of life. [12] *FACT-C*, Functional Assessment of Cancer Therapy-Colorectal. Physical well-being, Social/Family well-being, Functional well-being and Colorectal Cancer disease-specific subscale score range 0–28. Emotional well-being subscale score range 0–24. Total FACT-C Quality of Life score range 0–136. Total FACT-C trial outcome index score range 0–84. Higher scores indicate better quality of life

At baseline, survivors reported moderate overall scores for their experiences with cancer care, particularly in receiving timely appointments and care, as well as in the care team’s support for managing the effects of cancer and its treatments (56.8/100 and 53.1/100, respectively). Around two-thirds of survivors (64.5%) reported that their family members/friends were not involved in care discussions and perceived moderate quality of overall communication from the cancer care team (68.4/100). For baseline QOL, both survivors of lung and CRC reported lower emotional (mean = 17.8/24 and 19.0/24 respectively) and functional well-being scores (mean = 18.7/28 and 20.4/28, respectively). Total QOL scores ranged from 101.1/136 to 105.5/136. Of note, survivors of lung cancer reported lower QOL for physical well-being, emotional well-being, functional well-being, and total QOL as compared to colon cancer survivors.

Overall, PCPs were somewhat (55%) confident in their knowledge related to surveillance testing for cancer recurrence, late and long-term physical and psychosocial effects of treatment, and their role in survivorship care (Table 3). They reported high confidence in their knowledge related to communication with oncologists and patients on their role in survivorship care. However, only 54% of PCPs perceived that they have the skills necessary to provide survivorship care, while 34% disagreed.

**Table 3:** Primary care provider confidence about knowledge of cancer survivorship care (*N* = 75) [1]

Appropriate surveillance testing to detect recurrent cancer	
Not at all confident	1 (1.45%)
Somewhat confident	38 (55.07%)
Very confident	30 (43.48%)
*Missing/refused to answer*	*6*
**Long-term and late physical adverse effects of cancer and cancer treatment**	
Not at all confident	9 (12.86%)
Somewhat confident	40 (57.14%)
Very confident	21 (30%)
*Missing/refused to answer*	*5*
**Potential adverse psychosocial outcomes of cancer or its treatment**	
Somewhat confident	36 (52.94%)
Very confident	32 (47.06%)
*Missing/refused to answer*	*7*
**Which part of a patient’s survivorship care that I am responsible for**	
Not at all confident	8 (11.76%)
Somewhat confident	37 (54.41%)
Very confident	23 (33.82%)
*Missing/refused to answer*	*7*
**How to communicate with the oncology specialist team**	
Not at all confident	5 (7.14%)
Somewhat confident	31 (44.29%)
Very confident	34 (48.57%)
*Missing/refused to answer*	*5*
**How to communicate with patients on my role and responsibilities in their care after cancer treatment**	
Not at all confident	3 (4.29%)
Somewhat confident	31 (44.29%)
Very confident	36 (51.43%)
*Missing/refused to answer*	*5*
**Primary care providers (PCPs) have the skills necessary to provide follow-up care related to the effects of cancer or its treatment for survivors**	
Strongly/somewhat disagree	24 (34.29%)
Neither agree nor disagree	8 (11.43%)
Strongly/somewhat agree	38 (54.29%)
*Missing/refused to answer*	*5*
**PCPs have the skills necessary to initiate appropriate screening or diagnostic work-up to detect recurrent cancer, for survivors**	
Strongly/somewhat disagree	12 (17.14%)
Neither agree nor disagree	7 (10%)
Strongly/somewhat agree	51 (72.86%)
*Missing/refused to answer*	*5*
**PCPs are able to collaborate with oncologists to provide post-treatment care for survivors**	
Strongly/somewhat disagree	9 (12.86%)
Neither agree nor disagree	5 (7.14%)
Strongly/somewhat agree	56 (80%)
*Missing/refused to answer*	*5*

[1] *SPARCCS*, Survey of Physician Attitudes Regarding the Care of Cancer Survivors

**Fig. 1:**
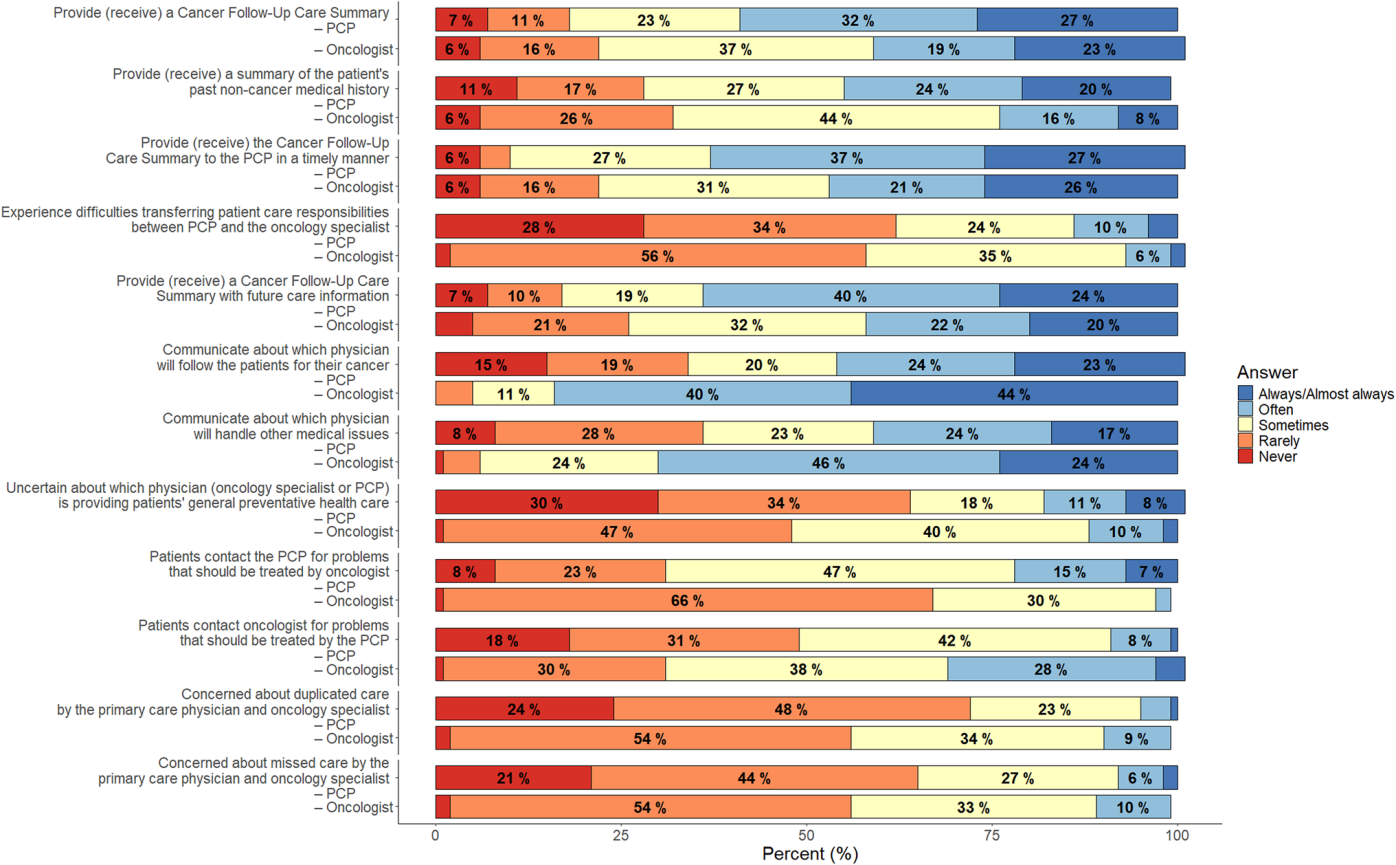
Primary care provider and oncologists perception of care coordination and communication (PCP *N* = 75; oncologist *N* = 129)

Primary care provider and oncologist’s perceptions of care coordination and communication are depicted in Fig. 1. PCPs reported that they often received cancer follow-up summaries from the oncology team (32%) and sometimes received past non-cancer medical history for survivors (27%). Conversely, oncologists reported that they sometimes (37%) provided a cancer follow-up care summary to PCPS, and sometimes (44%) provided survivors’ past non-cancer medical history to PCP. PCPs and oncologists reported rarely having trouble transferring care responsibilities in a timely manner (34% PCPs, 56% oncologists). There was also little uncertainty about which clinician would provide primary care, or whether patients received duplicate or missed care. Yet there was variation in perceptions between clinicians and the extent of clear communication about who would oversee which aspects of care. This is demonstrated by the following scenario, for example, oncologists sometimes furnished cancer-care summaries for PCPs and often provided future care information. Conversely, PCPs often received care summaries but sometimes obtained future care information from oncologists. Most oncologists (44%) reported they always provided patients with guidance about which clinician would oversee their cancer-related care, whereas the majority of PCPs reported (24%) often communicating this information. Lastly, there was some discord about how often patients directed care concerns to PCPs instead of oncologists. Most PCPs (47%) perceived patients contacted them for oncology-related concerns more often than oncologists.

## Discussion

The study was designed to examine the impact of an intervention that targeted patients, PCPs, and oncologists to engage in shared survivorship care for persons with a history of lung and CRC. The trial opened shortly after the start of the COVID-19 pandemic, and as a result, telehealth was used to facilitate the successful recruitment and enrollment of lung and CRC survivors. Recruitment data supports the feasibility of conducting complex interventions within an integrated oncology care system in Southern California and across multiple counties. Participant characteristics were largely representative of the target populations, with slightly older mean age for lung cancer (66) and younger for CRC (58). With increasing incidence rates of early-onset CRC, the number of younger survivors living with a history of CRC is likely to increase as well. Race and ethnicity distribution of the enrolled population mirrors the cancer center’s catchment area. The centralized administration of the intervention promoted high-quality intervention fidelity monitoring, and effective interventionist training, and enhanced the potential scalability of the intervention.

The baseline findings provide an illustration of the QOL for survivors of lung and CRC. Most patients denied difficulty managing their day-to-day activities secondary to their illness, but reports were highest among lung cancer survivors. Additionally, although survivors reported moderately high independence and physical function, data indicates there are areas of opportunity to securing timely care, appointments, and support in managing the effects of cancer treatment. This data aligns with previous evidence of ongoing QOL and care coordination challenges for lung and CRC survivors [21–23].

Clear, ongoing communication between providers is essential for effective survivorship care, and poor communication interferes with the development and implementation of quality care [24]. During the study, there were reported variations in perceived communications between providers, which raises concern about the congruence between the information relayed and information received. Thus, our data indicates there is a clear opportunity for improved communcation between PCPs and oncologists, which could improve patient care.

### Limitations

This study opened at the beginning of the COVID pandemic, and the sample of PCPs and oncologists was small. Subsequently, there were challenges associated with engaging PCPs particularly during the height of the pandemic. Lastly, our results are limited to Southern California and may not be generalizable to regions outside of the Greater LA region.

## Conclusion

Survivors of lung and CRC have complex care needs, yet evidence-based survivorship shared care models that effectively support patients, families, and providers remain limited. In addition, the effectiveness of oncology care teams in collaborating in the clinical setting has significant implications for patient care and outcomes [25]. Our results provide evidence that there are ongoing opportunities for improving healthcare delivery related to cancer survivorship care coordination and communication. Looking ahead, results on the efficacy of the lung and CRC survivor self-management intervention trial are pending.

### Implications for cancer survivors

Our findings suggest that persistent challenges remain for survivors of lung and CRC in accessing critical interventions needed to support and improve their QOL. There are opportunities to improve care coordination and communication among survivors, PCPs, and oncologists. Sustaining high-quality cancer survivorship care is essential for enabling cancer survivors to successfully navigate and actively participate in their care.

## Data Availability

The datasets generated during and/or analyzed during the current study are available from the corresponding author on reasonable request and based on National Cancer Institute policies.
